# How informed is informed consent?—Evaluating the quality of informed consent among surgical patients in a tertiary care hospital in Nepal

**DOI:** 10.1371/journal.pone.0288074

**Published:** 2023-07-10

**Authors:** Sunil Basukala, Oshan Shrestha, Niranjan Thapa, Sagun Karki, Ayushma Pandit, Bikash Bikram Thapa, Anup Thapa

**Affiliations:** 1 Department of Surgery, Nepalese Army Institute of Health Sciences, Kathmandu, Nepal; 2 College of Medicine, Nepalese Army Institute of Health Sciences, Kathmandu, Nepal; Imam Abdulrahman Bin Faisal University, SAUDI ARABIA

## Abstract

**Background:**

Informed consent-taking is a part of clinical practice that has ethical and legal aspects attached to it. This protects the autonomy of the patients by providing complete information regarding the rationale, modality, potential risks, benefits, and alternatives of the planned procedure to the patients. This enables the patients to make the right decision for themselves and their care. This study aims to find out if the informed consent-taking process has ensured the active participation of the patients or the next of kin in the decision-making.

**Materials and methods:**

This is a prospective cross-sectional study conducted in a military healthcare institution among patients undergoing major surgical procedures from July 2022 to October 2022. Ethical clearance was obtained before the commencement of this study. A structured questionnaire was prepared, and the collected data was refined in Excel and imported into SPSS for analysis.

**Results:**

A total of 350 individuals of mean age 47.95 ± 16.057 years were part of this study. The majority of the respondents were married, literate, and family by beneficiary category. All of the respondents received and signed the consent form. About 77% of the respondents read it completely, and 95.4% of them reported that it was understandable. The majority of the patients did not know who was going to perform the surgery, the alternatives to the planned treatment, the benefits of the surgery, or the outcome of non-treatment. On the patient satisfaction scale, 16.28% of the participants agreed that they were satisfied with the informed consent-taking process.

**Conclusion:**

Deficiencies in the informed consent-taking process were the lack of dissemination of adequate information on the nature, duration, pros and cons, post-operative state, and alternative of the planned procedure. A well-structured format of the consent form that is specific to a particular procedure should be adopted, and various alternatives to it must be disseminated to the patient or the next of kin to improve the quality of the informed consent-taking process.

## Introduction

Informed consent, which is taken before most medical procedures, is an integral part of clinical practice and has not only an ethical aspect but also a legal aspect attached to it to protect the autonomy of the patients [1]. This becomes more common and important in surgical practice to provide patients with the opportunity to decide for themselves before the operative procedure. Active involvement of the patient in the decision-making process becomes more important in the surgical field due to the invasive nature of the procedures, the use of anesthesia, cost factors, and a longer recovery period. The surgical informed consent-taking process has been established as an element of providing standard surgical patient care. The basic components recommended by the Nuremberg and Helsinki code, the U.S. National Institute of Health and the World Health Organization are the ‘assessment of preconditions’, ‘provision of information’, and the ‘stage of consent’ [2–5]. Application of these components into clinical practice enables clinicians to help their patients learn about the rationale, modality, potential risks, benefits, and alternatives of the planned procedure. A standard informed consent process helps the patient consider and reconsider what is important or best suited to them, enabling the patient to make the right decision for themselves regarding his or her care [6–8].

However, studies point out that the real practice is far from ideal. Taking informed consent from the patients hastily and minutes before the procedure when patients are not in the right state of mind to process complex information has been a major issue [9, 10]. The autonomy of the patients, being the main aim of the informed consent-taking process, is safeguarded only when complete information is disseminated understandably. Studies have identified different barriers and deficiencies that have played a negative role in achieving what is closer to an ideally informed consent-taking process. Improvement in the informed consent-taking process can be achieved only through the recognition of these barriers and taking prompt action against them [11–14]. The objectives of this study are to study the quality of the informed consent-taking process, patient satisfaction with this process, and potential deficiencies that may cause low patient satisfaction. This study aims to find out if the informed consent-taking process has ensured the active participation of the patients or the patient party in the decision-making.

## Materials and methods

This study is in line with STROBE guidelines [15].

### Ethical consideration

Ethical clearance for this study was obtained from the Nepalese Army Institute of Health Sciences Institutional Review Committee **(**Ref No: 641**).** Respondents to this study were informed about the nature of the study, and their consent was obtained before data collection.

### Study design and setting

This was a prospective cross-sectional study conducted in a military healthcare institution among patients undergoing major surgical procedures (both elective and emergency). The institution where this study is based is a tertiary-level hospital where approximately hundred major surgeries are performed in average each month. This study spanned four months, from July 2022 to October 2022. Patients of age more than eighteen and undergoing major surgical procedures (major surgical procedures included surgeries that had to be performed under regional or general anesthesia and needed post-operative evaluation) were part of this study, while exclusion criteria for this study were patients of age less than 18 years, referral cases, patients transferred to the intensive care unit after the surgery, readmission cases, and those not giving consent.

### Sample size and sampling technique

The total population sampling method was applied in this study, and all the patients undergoing major surgical procedures were assessed for eligibility. Patients meeting the inclusion criteria and presenting at our institution during the study period were all included in this study after receiving their consent. In the study period of four months, we encountered a total of 408 patients undergoing major surgical procedures. Those patients were compared against the inclusion and exclusion criteria of the study, out of which 350 patients met the criteria and were included in this study.

### Study tool

A structured questionnaire was made for the collection of data. The questionnaire was developed after a rigorous literature review of national and international guidelines on the informed consent-taking process. The tool was revised after the peer review. The study tool was then pretested and the study tool was again reformed according to the suggestions received. This tool included a section for demographic details, seven questions on the nature of the informed consent form, thirteen questions on the components of the informed consent form, and nine questions on the type of information that was present in the informed consent form. This study tool also included a 5-point Likert item to which the respondents responded how satisfied they were with the informed consent-taking process. The patients were handed over the questionnaire in the waiting room of the Operating Theatre (OT) just before they were taken inside the OT in elective cases. Whereas the data was collected after the surgery in emergency cases. The study tool is available as S1 File.

### Variables

Independent variables of this study were age, sex, address, marital status, literacy state, and beneficiary state of the hospital. While the other sections of the study tool that asked questions about the nature, components, and contents of informed consent and patient satisfaction were the dependent variables of this study. As this study was based in a military hospital, the patients were grouped according to their army rank or their beneficiary state. All the commissioned officers from and above Lieutenant were grouped as Officers; below the rank of Lieutenant and above Sergeant were grouped as Junior Commissioned Officers (JCO); the ranks from and below Sergeants were grouped as Other Ranks (OR), and retired personnel and family members who are eligible to receive healthcare were grouped as Family.

### Analytical strategy

The frequency was calculated along with the percentage for all the dependent and independent variables. The continuous variable was expressed in terms of mean and standard deviation. The 5-point Likert item that assessed patient satisfaction was treated as an ordinal variable, and ordinal regression was carried out by keeping it against independent variables. The 5-point Likert item that assessed patient satisfaction was treated with non-parametric test, for which the Mann-Whitney U test (for variables that had two categories) and the Kruskal-Wallis H test (for variables that had more than two categories) test were used to see if any differences existed among the responses of the patient satisfaction scale and categories of independent variables. Statistically significant variables were then treated with ordinal regression to find out the relationship between the variables. Data analysis was performed using SPSS software version 20.

## Results

This study had a total of 350 respondents with a mean (± standard deviation) age of 47.95 (± 16.057) years and a male: female ratio of 1.17:1. The majority of the respondents were married, literate, and family by beneficiary category. Among the types of surgical procedures, patients undergoing elective surgery were more common. The demographic details are given in Table 1.

**Table 1:** Demographic details.

Variables	Sample group (N = 350)	
	n	%
**Sex**		
** Male**	189	54.0%
** Female**	161	46.0%
**Marital state**		
** Married**	331	94.6%
** Unmarried**	19	5.4%
**Literacy**		
** Illiterate**	108	30.9%
** Literate**	242	69.1%
**Beneficiary Category**		
** Officer**	4	1.1%
** JCO**	31	8.9%
** Other ranks**	59	16.9%
** Family**	256	73.1%
**Type of Surgery**		
** Elective**	295	84.3%
** Emergency**	55	15.7%
**Health Care Cost**		
** All cost covered by the Military**	350	100%

This study explored the nature, components, and contents of the informed consent form. All of the patients had received an informed consent form before the surgical procedure which was in the native language. More than three-fourths of the respondents have reported reading the form and the form being understandable to them. The time of delivery of the consent form was some hours before the surgery as per the response of the majority of the respondents. About 31% of the participants were informed on which surgeon was going to operate, while the majority of them were informed about the nature of the surgery, benefits of the surgery, type of anesthesia being used, and given adequate time to ask questions and to sign the form. But only 10% of patients were given an alternative option other than the targeted one. Details of the responses to the questions related to the nature, components, and contents of the informed consent form are given in Tables 2–4 respectively.

**Table 2:** Responses to the questions on the nature of the informed consent form.

Information on	Sample group (N = 350)	
	n	%
**Have u received any written Informed consent form?**		
** Yes**	350	100%
** No**	-	-
** I don’t know**	-	-
Who signed it?		
** Patient**	79	22.6%
** Patient and relative**	110	31.4%
** Relative**	161	46.0%
**Did you read it?**		
** Yes**	272	77.7%
** No**	71	20.3%
** Partially**	7	2.0%
**Was It understandable?**		
** Yes**	334	95.4%
** No**	11	3.1%
** Partially**	5	1.4%
**Was it in Native language?**		
** Yes**	350	100.0%
** No**	-	-
**Time of delivering consent form**		
** Immediately before the surgery**	58	16.6%
** Some hours before the surgery**	172	49.1%
** The day before the surgery**	120	34.3%
**Who delivered it?**		
** Operative surgeon**	44	12.6%
** Nurse**	240	68.6%
** Administration**	66	18.9%
** I don’t know**	-	-

**Table 3:** Responses to the questions on the components of informed consent form.

Components of informed consent	Sample group (N = 350)		
	Yes	No	Do not remember
**Received information on who would perform the operation**	109 (31.1%)	230 (65.7%)	11 (3.1%)
**Told the diagnosis**	336 (96%)	14 (4%)	-
**Informed about the type/nature of the surgery**	232 (66.3%)	114 (32.6%)	4 (1.1%)
**Told the estimated duration of the surgery**	53 (15.1%)	288 (82.3%)	9 (2.6%)
**Informed about the benefits of the surgery**	213 (60.9%)	116 (33.1%)	21 (6.0%)
**Told the consequences of not undergoing the surgery**	168 (48.0%)	161 (46.0%)	21(6.0%)
**Received an explanation about the risks of the treatment**	185 (52.9%)	156 (44.6%)	9 (2.6%)
**Wanted more explanation on these risks**	157 (44.9%)	193 (55.1%)	-
**Received an explanation about alternative options for this treatment**	35 (10.0%)	311 (88.9%)	4 (1.1%)
**Told about the type of anesthesia to be used**	178 (50.9%)	161 (46.0%)	11 (3.1%)
**Got information on potential follow-up treatment (medical/surgical)**	61 (17.4%)	289 (82.6%)	-
**Given adequate time for decision to sign on the informed consent form**	267 (76.3%)	83 (23.7%)	-
**Given opportunity to ask questions**	240 (68.6%)	94 (26.9%)	16 (4.6%)

**Table 4:** Responses to the questions on the contents of the informed consent form.

Characteristics	Sample group (N = 350)	
	Yes	No
**Diagnosis**	52 (14.9%)	298 (85.1%)
**Type of surgery**	43 (12.3%)	307 (87.7%)
**Prognosis**	36 (10.3%)	314 (89.7%)
**Post- operative progress**	36 (10.3%)	314 (89.7%)
**Benefits of surgery**	38 (10.9%)	312 (89.1%)
**Outcome of non-treatment**	36 (10.3%)	314 (89.7%)
**Alternatives to proposed surgery**	16 (4.6%)	334 (95.4%)
**Chances of success of the surgery**	4 (1.1%)	346 (98.9%)
**Potential complications of the surgery**	346 (98.9%)	4 (1.1%)

On asking the participants to respond on their satisfaction level with the information they received during signing the informed consent form, almost three-fourths of the patients were neutral to it. The percentage of people who were satisfied was a higher than those who were dissatisfied. Details of the responses are given in Table 5.

**Table 5:** Patient satisfaction scale.

Satisfaction Scale	Strongly disagree	Disagree	Neutral	Agree	Strongly agree
**I am satisfied with the overall informed consent taking process**	8 (2.28%)	27 (7.71%)	258 (73.71%)	39 (11.14%)	18 (5.14%)

In order to study the differences in the patient satisfaction level among various groups of independent variables, the Mann-Whitney U test (for sex, literacy, marital status, and type of surgery) and the Kruskal-Wallis H test (for the beneficiary category) were applied. Literacy (U = 11229.00, p = 0.007), type of surgery (U = 4281.00, p = <0.001), and beneficiary category (χ2 = 4281.00, p = 0.018) yielded statistically significant values, suggesting that patient satisfaction varied among the various groups of these three variables. Details of the tests are available as S2 File.

The statistically significant variables were then treated by ordinal regression with age as a covariate. The model was statistically significant (χ2 = 85.279, p = <0.001) which suggests that there is a significant improvement in fit in the final model compared to the intercept-only model. Further, the Pearson chi-square test yielded a significant value (χ2 = 6546.76, p = <0.001) and the Deviance chi-square test yielded a non-significant value (χ2 = 405.59, p = 1.00). The overall result suggests that the model fits well.

The result of ordinal regression suggested that illiterate respondents were at 2.963 times higher odds of being satisfied compared to the literates, officers were at 49.764 times higher odds of being satisfied compared to the family, and the elective surgery group was at 0.06 times lower odds of being satisfied compared to the emergency surgery group. While the sex variable did not yield a statistically significant result. The details of ordinal regression are given in Table 6.

**Table 6:** Results of ordinal regression.

Variables	Coeff	S.E.	Wald	df	p	OR	95% C.I	
							Lower	Upper
**Age**	.002	.008	.062	1	0.803	1.006	0.987	1.018
**Literacy**								
** Illiterate**	1.086	.319	11.58	1	0.01	2.963	1.585	5.537
** Literate**	Reference							
**Beneficiary category**								
** Officer**	3.907	1.05	13.63	1	<0.001	49.764	6.256	395.85
** JCO**	-0.228	.461	.244	1	.621	.796	.323	1.965
** Other ranks**	0.567	.371	2.344	1	.126	1.764	.853	3.647
** Family**	Reference							
**Type of surgery**								
** Elective**	-2.821	.344	67.066	1	<0.001	.060	.030	.117
** Emergency**	Reference							

## Discussion

In this study, all the patients received written informed consent, which was signed by either the patient or their relative or both, and more than two-thirds of the patients agreed that they read it. But the act of reading and signing the consent form is meaningless unless the information that is divulged is complete and the patient is given enough time to register and reflect on that information. In order to assess this, patients were asked about time of the delivery of the consent form, and 49.1% of them responded that it was delivered only a few hours before the surgery. Also, only 12.6% of the participants received the consent form from the operating surgeon. On the questions that assessed the completeness of the informed consent form, almost all of the participants (96%) knew about their diagnosis; the majority of them knew about the nature of the studies (66.3%), the benefits of the surgery (60.9%), the risk of treatment (52.9%), and the anesthesia to be used (50.9%). But only 15.1% were told about the estimated time of surgery, and 10.0% were counseled regarding the alternative option to the planned procedure. Although 68.6% of the patients agreed that they were given an opportunity to ask questions, it is a matter of great discussion if the patient’s autonomy was really safeguarded. The concept of decision-making in health care has changed from a paternalistic view (where healthcare providers dictate to patients what to do by assessing what is best for them) to shared decision-making (the active participation of patients in decision-making) [16]. The reason for the low number of patients knowing about the alternative option may be due to the persistence of such a paternalistic view of decision-making, as shown by AlHaqwi et al. [17]. Most of the patients received an informed consent form just hours before the procedure, through persons other than operating surgeons, with information regarding the procedure planned by the surgeon but no information on the alternatives to that procedure. This reflects the practice of decision-making being more on the healthcare provider’s side than the patient’s side. The medical field has transitioned from doctors making the best decision for their patients to patients making their own decisions on their health, even if the experts do not see it as the best decision and the law supports this [18].

This study also assessed the patient-reported satisfaction level with the overall informed consent-taking process. Almost three-fourths of the patients were neutral toward it. The proportion of people who were satisfied was a little higher than that of those who were dissatisfied. The responses of the participants on the questions related to components of the informed consent form indicated substandard quality, but the patient satisfaction scale showed that only about 10% of the patients were not satisfied with the informed consent-taking process. This may be due to the population characteristics (health literacy state) of the country and the free healthcare provided by the military hospital. The country’s literacy rate is 65.9% (2011 AD data), and the literacy rate of this study was 69.1% [19]. Regression analysis showed that illiterate respondents had 2.963 times higher odds of being satisfied compared to literate respondents. This might have occurred as people who are not literate are dependent upon others on various aspects, including their health decision-making, as their health literacy is also low. This is supported by the findings of the study conducted by Budhathoki et al. [20]. In this study, conducted in a military hospital, officers were at higher odds of being satisfied. This may be due to the better quality of healthcare and patient counseling that high-ranking officers receive. Further study is needed in this aspect to explore the responsible factors for this result.

This study showed that the informed consent-taking process at the study site has not successfully protected the autonomy of the patients as the majority of the patients did not know about the benefits of the study and the alternative to the planned procedure. Bottrell et al. in their hospital-based study conducted in the United States of America, Vučemilo et al. in their multicentric study conducted in Croatia, and Ezeome et al. in their multicentric study conducted in Nigeria found the same results of benefits and alternatives components being deficient in the consent form [8, 21, 22]. Also, a systematic review and analysis of 14 studies, that studied the quality of informed consent forms across various parts of the world, showed that those 14 included studies had deficiencies in the benefits and alternatives part [23]. To counter this, various measures are to be taken with immediate effect. Healthcare institutions need to adopt strict regulations and the format of the consent form with a timely review; health care providers should be trained and made aware of the changing practice of decision-making; and a greater intervention through the government level and the medical council level is needed to inform patients regarding their rights. The Nepal Medical Council has ensured the autonomy of the patients and also recommends that basic information regarding diagnosis, procedure, anesthesia used, and probability of complications be included in the informed consent-taking form [24]. Regmi et al. have recommended incorporating training in ethics at the postgraduate level to improve adherence to informed consent and ethics [25].

This study adopted the total population sampling method, which is a type of purposive sampling and not a probability sampling method; the results obtained may not be fully generalizable. As this single-centric study was conducted in a military hospital where health care costs are covered by the military for serving soldiers and their families, the data obtained from this study may not be generalizable. Also, in the military setting, there was a high probability of information bias, which was attempted to resolve by assuring the patient’s privacy and secrecy so that the patient could give their honest opinion. The study tool used in this study was face validated and revised after peer review and suggestions received after the pilot study but was not validated with statistical methods, which is one of the limitations of this study. The site of the data collection was the waiting room of the OT and the time of data collection was right before they were taken inside the OT. Whereas in case of emergency cases, the site and time of data collection were after they were shifted from the post-operative ward to the general ward. Due to this gap in consent taking process and data collection time, recall bias cannot be ruled out among the 55 participants who underwent emergency surgery.

## Conclusion

Deficiencies in the informed consent-taking process were the lack of dissemination of adequate information on the nature, duration, pros and cons, post-operative state, and alternative of the planned procedure. In order to safeguard the autonomy of the patients, these deficiencies must be addressed by a well-structured consent form that is specific to a particular procedure, and various alternatives to it must be disseminated to the patient and the next of kin.

## Supporting information

S1 File Study tool. (DOCX)

S2 File Details of test. (DOCX)

S1 Raw data (XLSX)
