# Supplementary material for: How informed is informed consent?—Evaluating the quality of informed consent among surgical patients in a tertiary care hospital in Nepal
Source: PLoS One. 2023 Jul 10;18(7):e0288074. doi: 10.1371/journal.pone.0288074 (PMC10332608; doi:10.1371/journal.pone.0288074)
Supplement: S1 File — (DOCX) [file pone.0288074.s001.docx]

**Study tool**

Date:

Sex.:

Address:

Age (in years):

Address:

Occupation:

Marital Status:

Education level:

Relation/Rank:

Health care cost (covered by):

| **Elective** | **Emergency** |
| --- | --- |

Type of surgical intervention (tick the correct ):

**Table 1. Information on the delivery, signing, reading and comprehensibility of the written IC form (Please tick the correct answer)**

| s.no | Questions |  |  |  |
| --- | --- | --- | --- | --- |
|  | Have u received any written Informed consent form | Yes | I don’t know | No |
|  | Who signed it? | Patient | Patient + relative | Relative |
|  | Did you read it? | Yes | No, lack of time | Partially |
|  | Was It understandable | Yes | Partially | No |
|  | Was it in Nepali? | Yes | No | I don’t remember |
|  | Time before surgery | Immediately | Some hours before | The day before |
|  | Who delivered it? | Operative surgeon | Nurse | Administration  I don’t know |

| **Components of informed consent** | **Yes** | **No** | **Do not remember** |
| --- | --- | --- | --- |
| Signed an informed consent form |  |  |  |
| Received information on who would perform the operation |  |  |  |
| Told the diagnosis |  |  |  |
| Informed about the type/nature of the surgery |  |  |  |
| Told the estimated duration of the surgery |  |  |  |
| Informed about the benefits of the surgery |  |  |  |
| Told the consequences of not undergoing the surgery |  |  |  |
| Received an explanation about the risks of the treatment |  |  |  |
| Wanted more explanation on these risks |  |  |  |
| Received an explanation about alternative options for this treatment |  |  |  |
| Told about the type of anesthesia to be used |  |  |  |
| Got information on potential follow-up treatment (medical/surgical) |  |  |  |
| Given adequate time for decision to sign on the informed consent form |  |  |  |
| Given opportunity to ask questions |  |  |  |

**Table 4. Type of information present in the consent form in details. (Please tick the correct answer)**

| Characteristics | Yes | No |
| --- | --- | --- |
| Diagnosis |  |  |
| Type of surgery |  |  |
| Prognosis |  |  |
| Post- operative progress |  |  |
| Benefits of surgery |  |  |
| Outcome of non-treatment |  |  |
| Alternatives to proposed surgery |  |  |
| Chances of success of the surgery |  |  |
| Potential complications of the surgery |  |  |

**Table 5. Patient satisfaction regarding IC (On the scale of 1-5)- (Please tick the correct answer)**

| satisfaction | Strongly disagree | Disagree | Neutral | Agree | Strongly agree |
| --- | --- | --- | --- | --- | --- |
| I am satisfied with the overall informed consent taking process |  |  |  |  |  |
