# Supplementary material for: How informed is informed consent?—Evaluating the quality of informed consent among surgical patients in a tertiary care hospital in Nepal
Source: PLoS One. 2023 Jul 10;18(7):e0288074. doi: 10.1371/journal.pone.0288074 (PMC10332608; doi:10.1371/journal.pone.0288074)
Supplement: S2 File — (DOCX) [file pone.0288074.s002.docx]

Mann-Whitney U

| **Analyses** | **Mann-Whitney U** | **Z value** | **P value** |
| --- | --- | --- | --- |
| Patient satisfaction * Sex | 15212.50 | -0.002 | 0.997 |
| Patient satisfaction * Literacy | 11229.00 | -2.721 | 0.007 |
| Patient satisfaction * Marital status | 2702.50 | -1.333 | 0.182 |
| Patient satisfaction * Type of surgery | 4281.00 | -7.195 | <0.001 |

Kruskall-Wallis H

| **Analyses** | **Chi- square** | **df** | **P value** |
| --- | --- | --- | --- |
| Patient satisfaction * Beneficiary category | 10.099 | 3 | 0.018 |
